# Performance evaluation of biodegradable polymer sirolimus and ascorbic acid eluting stent systems

**DOI:** 10.1007/s10856-022-06699-8

**Published:** 2022-10-29

**Authors:** Won-Il Jo, Ji-Hyun Youn, So-Young Kang, Dae-Heung Byeon, Ho-In Lee, Hyoung-Mo Yang, Jun-Kyu Park

**Affiliations:** 1 CG Bio Co. Ltd, Seoul, Korea; 2 Department of Cardiology, Ajou University School of Medicine, Suwon, Korea

## Abstract

The purpose of this study was to evaluate the performance of biodegradable polymer sirolimus and ascorbic acid eluting stent systems with four commercially available drug-eluting stents (DES). We investigated the characterization of mechanical properties by dimension, foreshortening, recoil, radial force, crossing profile, folding shape, trackability, and dislodgement force. Additionally, we identify the safety and efficacy evaluation through registry experiments. Each foreshortening and recoil of D + Storm® DES is 1.3 and 3.70%, which has better performance than other products. A post-marketing clinical study to evaluate the performance and safety of D + Storm® DES is ongoing in real-world clinical settings. Two hundred one patients were enrolled in this study and have now completed follow-up for up to 1 month. No major adverse cardiovascular event (MACE) occurred in any subjects, confirming the safety of D + Storm® DES in the clinical setting. An additional approximately 100 subjects will be enrolled in the study and the final safety profile will be assessed in 300 patients. In conclusion, this study reported the objective evaluation of DES performance and compared the mechanical responses of four types of DES available in the market. There is little difference between the four cardiovascular stents in terms of mechanical features, and it can help choose the most suitable stent in a specific clinical situation if those features are understood.

**Figure:**
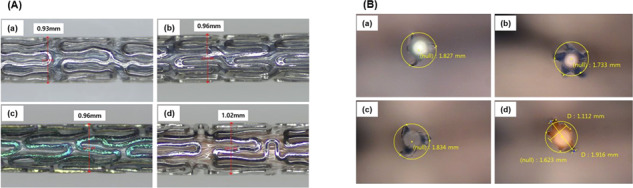
Graphical abstract

Graphical abstract

## Introduction

Coronary artery disease (CAD) is one of the leading causes of cardiovascular diseases such as angina pectoris, myocardial infarction, and ischemic heart failure, the leading cause of death worldwide [1]. The prevalence of CAD has been steadily increasing over the past decade due to changes in diet, lifestyle, and environment, and billions of dollars are spent annually on CAD treatment in the United States [2–4]. Treatment options for CAD include drug therapy, percutaneous coronary intervention (PCI), and coronary artery bypass grafting (CABG). Among them, PCI is a universal and non-invasive procedure to relieve the narrowing or occlusion of the coronary artery and improve blood supply to the ischemic tissue. PCI was applied to many lesions, such as bifurcation, calcified, and chronic total occlusion (CTO) lesions.

A stent used in PCI is a medical device, a tubular metal mesh inserted into a narrowed area of delivery systems, such as vessels, to normalize the flow of substances. Stents are classified into self-expandable and balloon-expandable types according to the expansion mechanism. In cardiovascular interventions, balloon-expandable stents that are strong enough to support blood vessels and have a relatively low metal content are commonly used [5]. Drug-eluting stent (DES) is coated drug on Bare-Metal Stent (BMS), a metal mesh, to prevent restenosis [6, 7]. According to ‘2022 Angioplasty Balloons Market Insights & Forecast 2021-2030’, DES held the most significant share of around 85.4% of the total market in 2020 with an overall value of approximately USD 6.95 Billion and is prevised to grow at a CAGR of 8.77% [8]. This study aimed to analyze the mechanical performance of the balloon-expandable DES.

The stent’s mechanical performances are crucial and valuable indicators of their clinical usefulness [9, 10]. Nevertheless, a paucity of data is available to compare mechanical performances in vitro. The mechanical properties of the stents, such as deliverability, radial force, recoil, foreshortening, and side branch access, are affected by their design and structure [11, 12]. To produce coronary stents, slotted tube stents are commonly used [13, 14]. However, existing slotted stents have high radial force, but low delivery potential, so more force is required to deliver them to the lesion, and because of their low flexibility, there is a possibility that they may not expand according to the shape of blood vessels [15]. To solve these problems, we reduced the crossing profile and optimized the overall structural design of the stent. Parameters for in vitro evaluation of the mechanical performance of DES include radial force, pushability, and trackability [16, 17]. This study aims to verify the mechanical performances according to the design change of the slotted stent using these parameters.

The stent investigated in this study is D + Storm® DES (CG Bio Co., Ltd, Seoul, Korea), a sirolimus-coated balloon expandable DES for coronary arteries. We will compare the mechanical performance of D + Storm® DES with four commercially available DES products and report interim results from an ongoing post-market clinical study.

## Materials and methods

### Materials

This study compared the mechanical performance of D + Storm® DES (CG Bio, Seoul, Korea) with those of three commercially available stents, Synergy™ (Boston Scientific, MA, USA), Xience sierra™ (Abbott Vascular, MN, USA), and Orsiro® (Biotronik, Berlin, Germany). The information for the four products is shown in Table 1. The most commonly used stent size, Ø2.75 × 18 mm, was used in this study, but only for Boston Scientific Synergy™, which does not produce that size; Ø2.75 × 20 mm was used. To evaluate the mechanical performance of the stents, thickness/width, foreshortening, recoil, radial force, crossing profile, folding shape, trackability, and dislodgement force were measured. For each parameter, values from 3 samples per group were averaged (*n* = 3).

**Table 1:** Characteristics of drug-eluting stent

Stent	Alloy	Sturt thickness (µm)	Polymer	Drug	Coating thickness(µm)	Stent platform
D + Storm	CoCr	75	PLA	Sirolimus	5 (Abluminal)	8-6-8 hybrid open cell
Synergy	PtCr	74	PLGA	Everolimus	4 (Abluminal)	SYNERGY
Orsiro	CoCr	60	PLLA with silicon carbide layer	Sirolimus	7.4 (Conformal)	Pro-kinetic Energy
Xience Sierra	CoCr	81	Permanent fluorinated polymer	Everolimus	7.8 (Conformal)	MULTI-LINK VISION

*CoCr* cobalt chromium, *PtCr* platinum chromium, *PLGA* poly(lactic-co-glycolic acid), *PLLA* poly-L-lactide, *PLA* Polylactic acid

The product’s thickness and width were analyzed by using SEM equipment (CX-200, COXEM, Daejeon, Korea), which was set up with acceleration voltage at 5 kV, emission current at 100 uA, and magnification at ×500. When the stent is cut directly, there is a possibility that the stent will be cut obliquely rather than vertically, In order to improve accuracy, the stent was molded, and then the mold was cut vertically for the analysis. We used a dimensional analysis program to measure dimensions and store photos. Before the observation, the products were sputter-coated with gold for 1 min.

### Crossing profile and folding shape

The crossing profile, which measures the diameter of a stent disposed on a balloon catheter, was measured by using a tool microscope (EGZV-745, EG Tech, Anyang, Korea). After fixing the stent to the tool microscope, the average diameter was calculated by measuring the outer diameters of both ends and the middle of the stent using a dimension measurement program of the tool microscope.

After expanding the drug-eluting stent at normal pressure (NP), it was held for 30 s. Then, 30 s after shrinking the balloon catheter, the stent was removed. The shape of the balloon catheter was then placed perpendicular to the lens and analyzed by using a tool microscope (BVMP-114C-04, Bestec vision, Gunpo, Korea).

### Foreshortening and recoil

The foreshortening and recoil were analyzed by using a tool microscope (CW-1501N, Chien Wei precise technology Co. Ltd, Kaohsiung, Taiwan).

In foreshortening, the balloon was expanded to its nominal pressure and maintained for 30 s. After fixing the transmission system to the toolmaker’s microscope, the length of the stent was measured (A). The stent length was measured 30 s after shrinking the balloon catheter (B). The foreshortening value was calculated by substituting the following test formula:

$${{{\mathrm{Foreshortening}}}}\,\left( \% \right){{{\mathrm{ = }}}}\frac{{{{{\mathrm{A - B}}}}}}{{{{\mathrm{A}}}}} \times {{{\mathrm{100}}}}$$

In recoil, the balloon was expanded up to its nominal pressure and maintained for 30 s. The outer diameter of each endpoint and the center of the stent were measured;(A) then, 30 s after the balloon catheter was shrunk, the outer diameter of each endpoint and the center of the stent were measured. (B) The value was calculated by substituting variables in the following test formula:

$${{{\mathrm{Recoil}}}}\left( {{{\mathrm{\% }}}} \right) = \frac{{A - B}}{A} \times 100$$

### Radial force

**Fig. 1:**
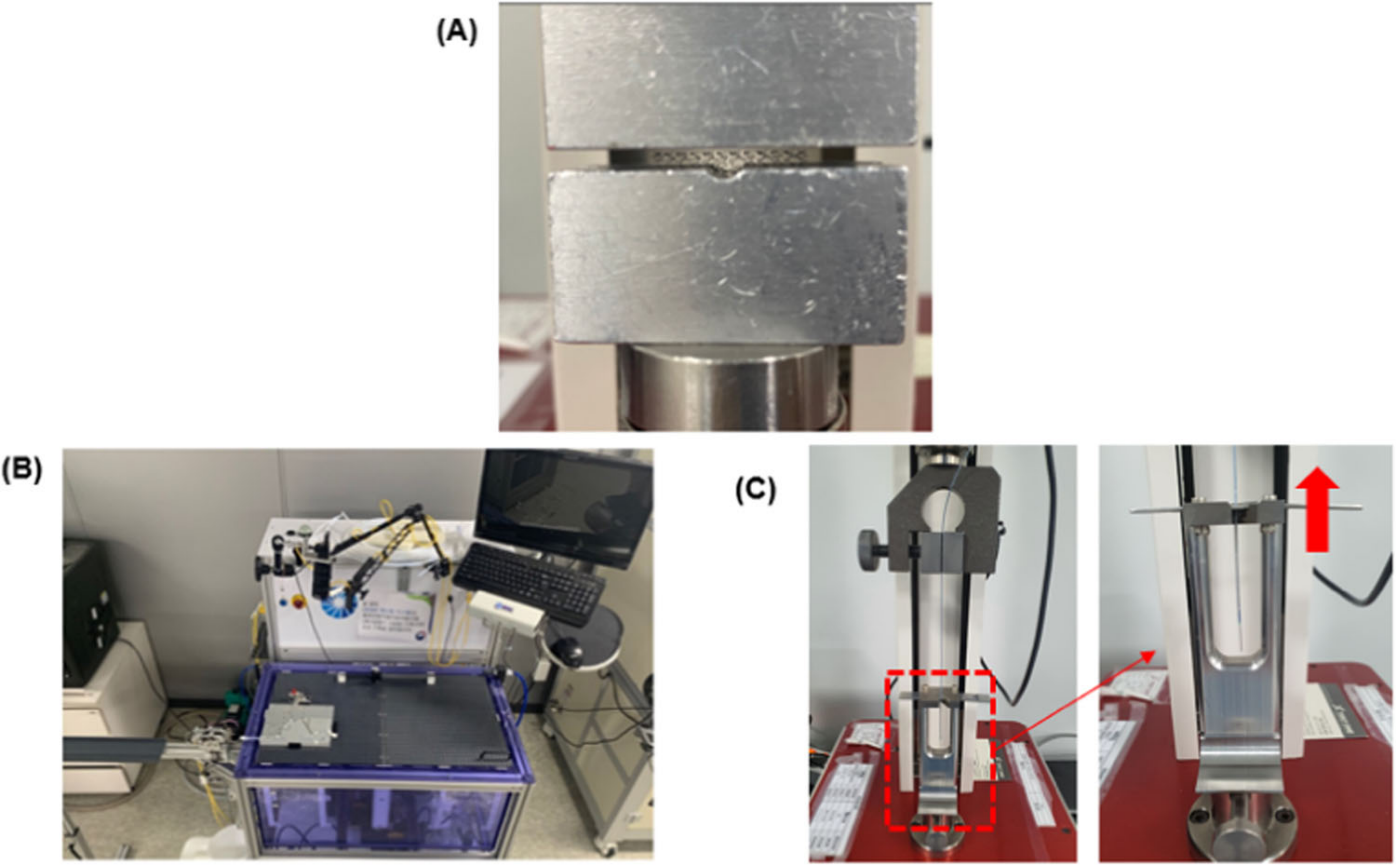
The instruments for testing radial force (**A**), trackability (**B**), stent dislodgment bench test (**C**)

The radial force was measured by using the universal testing machine (UTM, TO-101G, TEST ONE, Siheung, Korea). A fully-expanded stent was placed on the jig of equipment (Fig. 1A). The force required to compress the stent mounted on the jig to the radius was measured. A 10-N load cell was used, and the compression speed was 10 mm/s. The load cell continuously recorded the force when the stent was compressed and expanded.

### Trackability

The delivery force was measured by using the Interventional Device Testing Equipment (IDTE3000, Machine solutions, AZ, USA), a standardized pushability/trackability intervention medical device test equipment (Fig. 1B). The performance of IDTE3000 interventional medical devices can be quantitatively tested, compared, and recorded. This equipment is suitable for testing catheters, guidewire, stents, delivery systems, endoscopes, and scope tools. The jig for trackability testing was designed based on international standards [16, 17]. As a trackability measurement method, after calibration, the load cell is filled with water in the water tank and heated to 37.5 °C, and the water is circulated by using a pump. The model conducted in the experiment was placed in a tank, and the catheter was combined with the guidewire and then fixed to the roller. And then, trackability measured force when catheter insert target position through guide wire during.

### Dislodgement force test

The dislodgment force refers to the force required to dislodge the stent from the delivery system balloon completely. There are two categories of stent securement tests; guide-types and lesion-types, according to American Society for Testing and Materials (ASTM) F2394-07 recommendations (Standard Guide for Measuring Securement of Balloon Expandable Vascular Stent Mounted on Delivery System). We performed the shim test for the guide type. The stent delivery system is fixed to the UTM, and the proximal part of the stent is fastened to the holder of the “V” (diameter of 0.09 mm; Fig. 1C). The stent system was mounted in a UTM (TO-101G, Testone, Siheung, Korea) with a 50 N force load cell (measuring range, 0–50 N; accuracy, 0.1 N). The stents were dislodged from the proximal to the distal tip of the stent delivery system at 20 mm/min. The force at which the stent starts to dislodge relatively to the balloon was measured and recorded, and two forces were measured. The initial peak displacement force was defined as the first peak in force that occurs during or after stent displacement concerning the balloon, whereas the peak dislodgement force was defined as the peak or maximum force required to dislodge the stent from the delivery system balloon completely.

### Statistical analysis

All statistical analysis was accomplished by using GraphPad Prism (San Diego, CA, USA). One-way ANOVA with Tukey’s multiple comparison posttests was performed to compare the samples. The results considered no significant (ns) when *p* > 0.05 and statistically significance when *p < 0.05, **p < 0.01, and ***p < 0.001, and ****p < 0.0001.

## Evaluation method of clinical study

### Study design and patients

A real-world, prospective, multi-center, non-randomized, investigator-initiated, open-label clinical study to assess the effectiveness and safety of D + Storm® DES is ongoing. This study is expected to be from September 2020 to December 2022, and patients with coronary artery disease who underwent interventional procedures using D + Storm® DES in two institutions were enrolled in this study. Since the purpose of this study is to assess the real-world effectiveness and safety of the technique, the inclusion criteria were as follows: (1) age ≥19 years, (2) participants undergoing D + Storm® DES, (3) voluntary provision of participant’s informed consent. Patients who have undergone DES other than D + Storm® DES, whose expectancy was 12 months or less, who had a cardiogenic shock, whose left ventricular ejection fraction was <20%, who were pregnant or planned to conceive, who had to stop antiplatelet therapy within 12 months after enrollment were excluded in this study. This study was approved by the Institutional Review Board of the Ajou University Hospital and Inha University Hospital (AJIRB-DEV-OBS-20-251, 2020-09-001-000), and written consent was obtained from all study participants. For PCI, a standard interventional technique was used. In addition, direct and branch stenting were performed and other coronary artery interventional methods, including directional atherectomy and rotational atherectomy, could be performed according to the practitioner’s discretion. Clinical follow-up is conducted at 1, 6, and 12 months via telephone and outpatient visits.

### Study variables

Data on angina symptoms, re-hospitalization, revascularization, and significant ischemic clinical event occurrence are collected. Electrocardiogram and cardiovascular data (CPK, CK-MB, and Troponin) are also collected for patients with recurrent infarction. To determine the fractional flow reserve, intravascular ultrasound (IVUS) or optical coherence tomography is performed according to the practitioner’s discretion. The primary efficacy endpoint was defined as a composite event of death, myocardial infarction (MI), or target lesion revascularization (TLR). Secondary efficacy endpoints included all-cause death, cardiac death, MI, target vessel revascularization (TVR), TLR, Stent thrombosis, and procedure success rate.

### Statistical analysis

In this study, an interim analysis was performed on participants who had completed a 1-month follow-up among the enrolled subjects. Continuous variables were presented as mean values and standard deviations for baseline characteristics and result values, while categorical variables were expressed as frequencies and percentages. All statistical analyses were performed by using SAS® System Release 9.2 (SAS Institute, Cary, NC, USA).

## Results

### Thickness/width

The thinner the thickness and width of the stent, the smaller the profile during crimping, which is advantageous for entry performance [18]. The characteristics of DES are shown in Table 1. Only the cross-sectional shape (thickness/width) of the Xience sierra™ stent was square, and the other three were rectangular.

### Crossing profile and folding shape

**Fig. 2:**
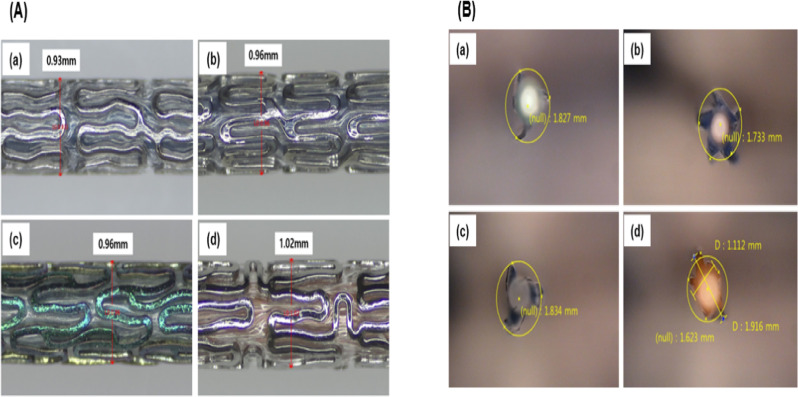
Crossing profile analysis result (**A**) and folding shape (**B**) of D + Storm® DES (Drug eluting stent) (a), Synergy^™^ (b), Orsiro® (c), and Xience sierra^™^ (d)

When the stent and balloon catheter are combined, the small crossing profile may allow smoother access to the desired lesion (even in small blood vessels). The crossing profile values for D + Storm® DES, Synergy™, Orsiro^®,^ and Xience sierra™ were 0.93, 0.96, 0.96, and 1.02 mm, respectively. D + Storm® DES had the smallest value (0.93 mm), followed by Synergy™ and Orsiro® products (0.96 mm both; Fig. 2A).

If the deflated balloon catheter is flat, it may lead to migration of the deployed DES and may cause damage to blood vessels during removal. Increasing the number of wings in the final fold reduces the likelihood of flattening but increases the profile. Therefore, it is suggested that the folding shape is selected by considering the DES and balloon catheter profile. The final folded shape of the D + Storm® DES and Orsiro® has three wings, while the Synergy™ and Xience sierra™ have five wings (Fig. 2B). The folding shape of any balloon catheter was not flat.

### Mechanical properties (Foreshortening, recoil, radial force)

**Fig. 3:**
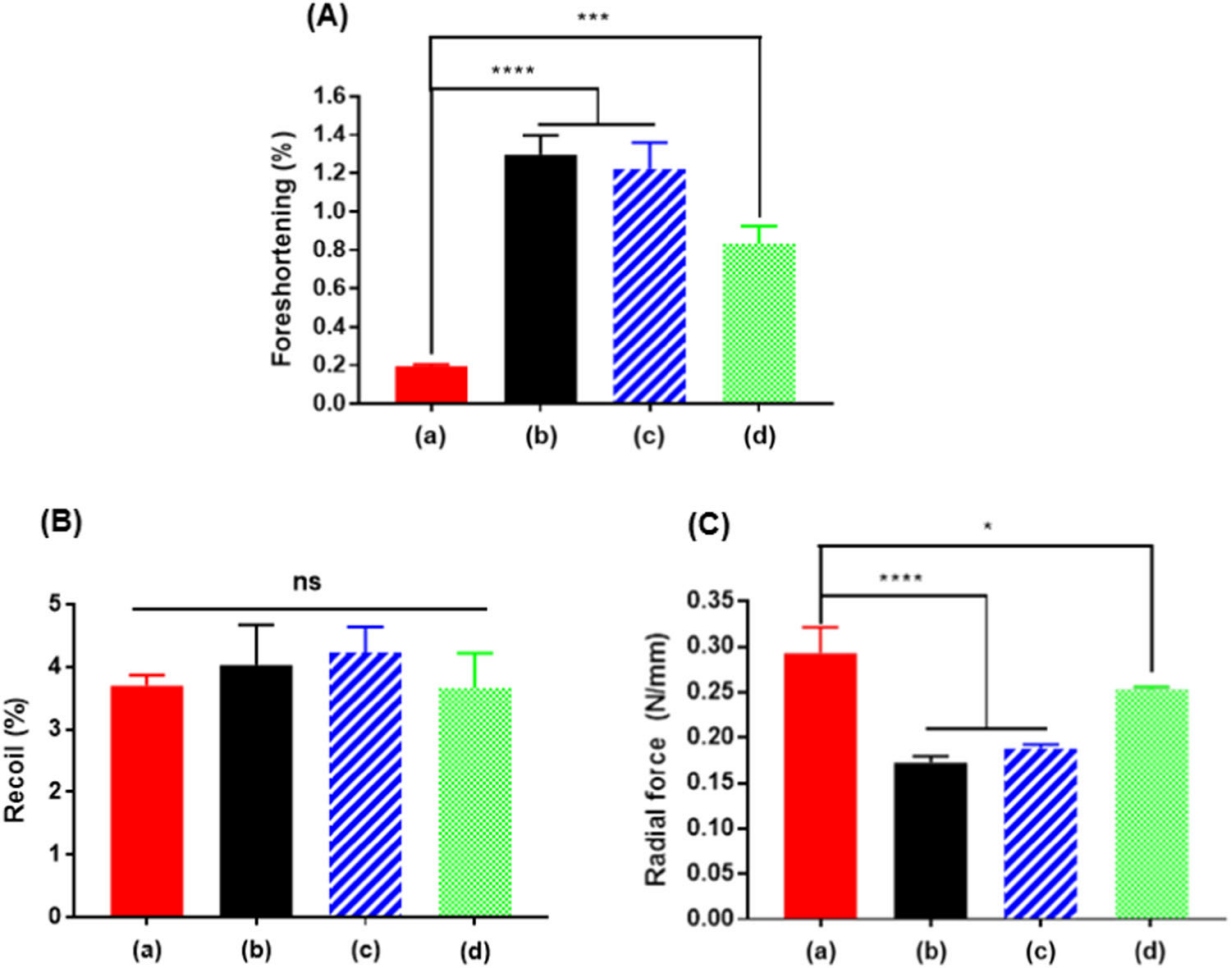
Mechanical properties of D + Storm® DES (Drug-eluting stent) (a), Synergy^™^ (b), Orsiro® (c), and Xience sierra^™^ (d). **A** Foreshortening, **B** Recoil, **C** Radial force analysis

Foreshortening, recoil, and radial force were evaluated as mechanical properties of stents. The result of foreshortening has shown 0.195, 1.297, 1.223, and 0.833% for D + Storm® DES, Synergy^™^, Orsiro®, and Xience sierra^™^, respectively (Fig. 3A). A smaller reduction range after stent expansion (=foreshortening) is advantageous during the procedure. Products with less than 1% foreshortening were D + Storm® DES and Xience sierra™. The result of recoil has shown 3.70, 4.03, 4.57, and 3.80% (D + Storm® DES, Synergy^™^, Orsiro®, and Xience sierra^™^, respectively; Fig. 3B), The stent with the lowest difference between the inflated diameter and the diameter after balloon catheter removal in recoil was D + Storm® DES. The result of the radial force has shown in Fig. 3C. D + Storm® DES, and Xience sierra^™^ had a considerably higher radial force (0.293 and 0.253 N/mm, respectively) than other products. All three mechanical properties of D + Storm® DES were overall favorable compared to other products.

### Trackability

**Fig. 4:**
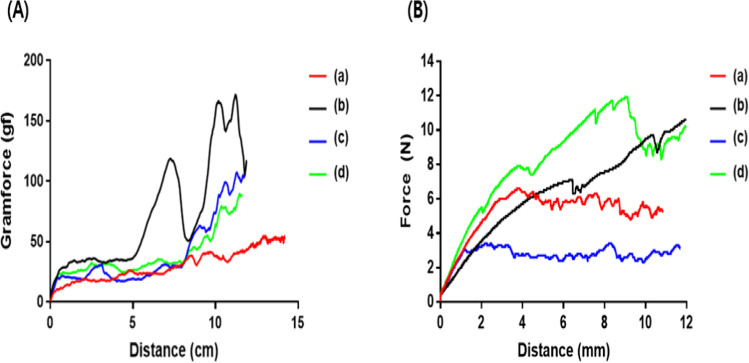
Trackability (**A**) and dislodgement force test (**B**) of D + Storm® DES (Drug-eluting stent) (a), Synergy^™^ (b), Orsiro® (c), and Xience sierra^™^ (d)

The traceability that is critical to the correct DES placement was tested in accordance with ASTM F 2394-07. D + Storm® DES, with the smallest crossing profile, showed the best performance (Fig. 4). The product with the best trackability performance was the D + Storm® DES with the smallest crossing profile, although a significant correlation between the crossing profile and trackability was not confirmed.

### Dislodgement force evaluation

The dislodgement force between the balloon catheter and DES was measured, which is important for the correct placement of the DES. The product that showed the greatest value was Xience sierra™, which had a structure that overlapped without interfering with each other when the cells of the stent collapsed. In the case of Synergy™ products with similar overlapping structures with Xience sierra™, the force continued to increase even after the initial dislodgement. On the other hand, Orsiro®, which has a structure that does not overlap each other when the cells of the stent are collapsed because the cells are connected in a bridge shape, showed the lowest value. D + Storm® DES showed a value between Xience sierra™ and Orsiro® values.

### Clinical results

Interim analyses of the clinical data of the enrolled subjects from September 2020 to August 2022 in two institutions in Korea were performed. One-month follow-up has been completed so far, and the baseline characteristics (demography, lesion, and procedure) of the 201 patients who received medical devices for clinical trials are outlined in Table 2. No patients developed MACE, all-cause death, cardiac death, myocardial infarction, target vessel/lesion revascularization, or stent thrombosis. Follow-up will be continued for up to 12 months (Table 3). Approximately 100 subjects will be enrolled in the study, and the final safety profile will be assessed in 300 patients.

**Table 2:** Baseline characteristics of subjects

	*N* = 201
Age (years)	64.43 ± 9.14
Sex, *n* (%)	
Male	141 (70.15)
Female	60 (29.85)
BMI (kg/m^2^)	24.64 ± 3.10
Current smoker, *n* (%)	56 (27.86)
Diabetes, *n* (%)	
No	128 (63.68)
Treated	73 (36.32)
Untreated	0 (0.00)
Hypertension, *n* (%)	
No	89 (44.28)
Treated	111 (55.22)
Untreated	1 (0.50)
Hyperlipidemia, *n* (%)	
No	109 (54.23)
Treated	87 (43.28)
Untreated	4 (1.99)
Onset diagnosis	1 (0.50)
Family Hx, *n* (%)	55 (27.36)
Prior MI, *n* (%)	13 (93.53)
Prior CABG, *n* (%)	2 (1.00)
Prior PCI, *n* (%)	39 (19.40)
Diagnosis, *n* (%)	
STEMI	10 (6.06)
NSTEMI	11 (6.67)
Unstable angina	109 (66.06)
Stable angina	35 (21.21)
Prior ischemic heart disease	0 (0.00)
Others	0 (0.00)
Target lesion, *n* (%)	
LAD	108 (53.73)
Lcx	38 (18.91)
RCA	34 (16.92)
LM	0 (0.00)
D	8 (3.98)
OM	8 (3.98)
RI	2 (1.00)
PDA	3 (1.49)
PLB	0 (0.00)
AHA/ACC classification, *n* (%)	
A	1 (0.50)
B1	50 (24.88)
B2	36 (17.91)
C	114 (56.72)
RVD (mm)	3.07 ± 0.41
Lesion length (mm)	25.70 ± 10.37
Stent length (mm)	26.15 ± 7.43
Stent diameter (mm)	2.98 ± 0.37

Data are presented as frequencies (%) or the mean ± standard deviation

*BMI* body mass index, *Hx* history, *MI* myocardial infarction, *CABG* coronary artery bypass grafting, *PCI* percutaneous coronary intervention, *STEMI* ST-elevation myocardial infarction, *NSTEMI* non ST segment elevation myocardial infarction, *LAD* left anterior descending, *Lcx* left circumflex artery, *RCA* right coronary artery, *LM* left main coronary artery, *D* diagonal branch, *OM* Obtuse marginal branch, *Ramus* ramus intermedius artery, *PDA* posterior descending artery, *PLB* posterolateral branch, *RVD* reference vessel diameter

**Table 3:** Interim-analysis results at 1-month follow-up

*n* (%)	*N* = 201
MACE	0 (0.00)
Death	
All-cause death	0 (0.00)
Cardiac death	0 (0.00)
MI	0 (0.00)
TVR	0 (0.00)
TLR	0 (0.00)
Stent thrombosis	0 (0.00)

Data are presented as frequencies (%)

*MACE* major adverse cardiovascular event, *MI* myocardial infarction, *TVR* target vessel revascularization, *TLR* target lesion revascularization

## Discussion

We conducted this study to compare the mechanical performances of the four DESs available in the market. There is no doubt that the situation is much more complex in the real world than in vitro. The mechanical performance of the DES can be explained as the combination of the size and physical characteristics of the stent and the force required to safely and easily deliver the stent to the target lesion. Common values used to explain the DES include the width, thickness, and crossing profile, and the stent should be placed in a correct location without damage on its way to the target lesion, and without dislodgement. Those are the basic preconditions for successful procedures. However, most of the technical data for each product are left to the user to guess because little technical data are disclosed, except for the user manual of the commercially available product. Accordingly, it is considered that in vitro simulation is vital to understand the physical properties of the stents and provide users with the best indicators.

During stent insertion, various factors, such as the length of the lesion, among other factors, should be taken into consideration when choosing the length of a stent. Among them, foreshortening is one of the most important factors in performing the procedure in a real-world setting, as this is an indicator of the shrink ratio when the balloon catheter shrinks from the expanded length to the loaded length. D + Storm® DES and Xience sierra^™^ showed the best performances of foreshortening among other products.

The recoil is a ratio of the difference between the diameter of the expanded stent and the diameter after the balloon of the stent has been removed. The appropriate stent is selected after measuring the diameter of the impacted vessel. When a stent with smaller in diameter than the marked value due to recoil, it fails to fit close to the vessel wall, resulting in its migration during blood flow. When comparing the degree to which the length decreases in diameter after placing the stents (recoil), the results were all analyzed within a range that did not have a significant clinical impact despite the differences between structures.

The radial force can check the extent to which the stent can maintain the shape of the blood vessel, even with vasoconstriction/relaxation. If the radial force is too strong, the vessel wall might be scarred; on the other hand, if it is too weak, the stent could migrate to other locations. Therefore, finding the appropriate force is crucial. D + Storm® DES and Xience sierra™ had a considerably higher radial force (0.293 and 0.253 N/mm, respectively) than other products. It was understood that D + Storm® DES and Xience sierra™ could fit closer to the vessel walls.

When this performance is confirmed, it is thought that the D + Storm® DES product will perform better than other product lines in terms of physical properties, just like in the in vitro experiment, through the pursued in-vitro experiment. In addition, since the force when compressed in a longitudinal form was analyzed, it is necessary to examine that when compressed in a cylindrical (radial) form to increase the reliability of the data.

As a result of the trackability test, the correlation cannot be determined entirely since there were several products with good approaching performance despite their thickness and width. Therefore, for the DES to reach each target lesion and be transplanted in optimal conditions, various conditions, such as the structure of the stent, balloon catheter profile, and clamping condition should be appropriately harmonized. Although it may differ from the clinical environment in which DES is directly inserted into blood vessels, D + Storm® DES products performed significantly better than others when tested according to ASTM standards for traceability and dislodgement testing.

In a previous study, D-Strom® DES demonstrated its pivotal clinical effectiveness and safety in patients with coronary artery disease [18]. In addition to mechanical performance, safety data is secured through post-marketing clinical trials to confirm stent performance in actual clinical situations. At a one-month follow-up of 201 enrolled patients, no MACE, all-cause death, cardiac death, myocardial infarction, target vessel/lesion revascularization, or stent thrombosis were reported. About 100 additional subjects will be enrolled in the future, and 300 patients will be evaluated for safety over 12 months.

Although there is a limitation that the in vitro and actual clinical environment may be different, the mechanical performance of the stent can be directly or indirectly evaluated through various indicators as in this study, in addition to the longitudinal strength of the stent reported through previous studies [19–21]. Utilizing these indicators will help to select the most suitable DES for a specific lesion in a particular situation in the future.

## Conclusion

In this study, the mechanical properties of a D + Storm® DES system made of a biodegradable polymer coated with sirolimus and ascorbic acid were compared to four commercially available types of DES. In addition, interim analysis results of post-marketing clinical studies demonstrating the clinical safety of the D + Storm® DES system have been reported. Understanding the mechanical properties of each DES will help select the optimal stent for specific clinical situations.
